# Comparison between mono-tacrolimus and mono-prednisone in the treatment of ocular myasthenia gravis: a retrospective, observational cohort study

**DOI:** 10.3389/fimmu.2026.1917837

**Published:** 2026-10-13

**Authors:** Ziling Wang, Jinna Li, Zhelu Fan, Luyang Ma, Jing Ma, Huiqiu Zhang, Menghan Su, Dan Liu, Huifen Wang, Juan Wang, Xueli Chang, Junhong Guo, Wei Zhang

**Affiliations:** 1 Department of Neurology, First Hospital of Shanxi Medical University, Taiyuan, China; 2 Research Center for Neurological Diseases, Shanxi Medical University, Taiyuan, China; 3 First Clinical Medical College, Shanxi Medical University, Taiyuan, China; 4 Department of Neurology, Jincheng People’s Hospital, Jincheng, China; 5 Department of Anesthesiology, First Hospital of Shanxi Medical University, Taiyuan, China

**Keywords:** efficacy, monotherapy, ocular myasthenia gravis, prednisone, tacrolimus

## Abstract

**Objective:**

The use of tacrolimus (TAC) in ocular myasthenia gravis (OMG) is generally reserved for cases refractory to prednisone (PRED); however, the advantage of monotherapy with tacrolimus (mono-TAC) over monotherapy with PRED (mono-PRED) remains unknown. The present study aimed to compare the efficacy and safety of mono-TAC and mono-PRED in OMG.

**Methods:**

This single-center, retrospective study analyzed collected data from adult patients with treatment-naive newly diagnosed OMG who received either mono-TAC or mono-PRED. The primary outcome was the time to reach minimum symptom expression (MSE, defined as MG-ADL ≤ 1), with time to MG-ADL score of 0 as an exploratory endpoint. Secondary outcomes included the mean changes in Myasthenia Gravis Activities of Daily Living (MG-ADL) and ocular Quantitative Myasthenia Gravis (O-QMG) scores from baseline to months 1, 6, and 12 after treatment, respectively, as well as the monotherapy retention rates at month 12 in the two groups. Safety was assessed based on medical records during the hospitalization to monitor the adverse events.

**Results:**

Forty-two patients were enrolled (mono-TAC: n = 27; mono-PRED: n = 15). For the primary outcome, the median time to MSE was 6.0 months (95% CI: 2.523-9.477) for PRED vs. 12.0 months (95% CI: 8.342-15.658) for TAC, with no significant difference between groups (log-rank P = 0.253). The exploratory endpoint of MG-ADL = 0 also showed comparable results between groups (P = 0.928). Secondary outcomes showed no significant differences in the mean changes in MG-ADL and O-QMG scores from baseline to 1, 6, and 12 months post-treatment (all P > 0.05). The monotherapy retention rates were similar (TAC: 81.5% vs. PRED: 73.3%, P = 0.698). Both treatments showed favorable safety profiles.

**Conclusion:**

In conclusion, no statistically significant difference was observed in treatment response between tacrolimus and prednisone.

## Introduction

Myasthenia gravis (MG) is an autoimmune disease, characterized by muscle weakness and fatigue (1). It is B-cell mediated, and is associated with antibodies directed against the acetylcholine receptor, muscle-specific kinase (MuSK), or lipoprotein-related protein 4 (LRP4) at the neuromuscular junction (2). Ocular MG (OMG) is frequently defined as patients who present with purely ocular symptoms at disease onset (3, 4). Of these, 50% to 80% develop generalized disease, most within two years (3).

Oral prednisone (PRED) is recognized as the first-line therapy for OMG (4). However, its long-term use is complicated by numerous adverse effects, and some patients show limited efficacy (5, 6). Nonsteroidal immunosuppressants, including TAC (TAC), cyclosporine A (CsA), azathioprine (AZA), mycophenolate mofetil (MMF), and methotrexate (MTX), are usually used in conjunction with PRED initially as steroid-sparing agents or when the response to corticosteroids is inadequate (4, 7–9). TAC and CsA inhibit lymphocyte activation by suppressing the calcineurin pathway (4). Their inhibition exhibits an onset of action comparable to oral PRED but faster than other immunosuppressants (7); thus, TAC or CsA monotherapy has the potential to become an induction regimen for OMG. Nephrotoxicity of CsA limits its use (4). Herein, we investigated the efficacy and safety of TAC monotherapy in naive OMG patients.

## Methods

### Participants and study design

This is a single-center, retrospective cohort study, conducted at the First Hospital of Shanxi Medical University, Shanxi Province, China. This study has been approved by the Institutional Ethics Committee of the First Hospital of Shanxi Medical University (KYLL-2025-354) and performed in accordance with the ethical standards laid down in the 1964 Declaration of Helsinki and its later amendments. The diagnosis of OMG was based on the following criteria: (a) typical fluctuating extraocular muscle weakness with positive fatigue and/or ice-pack test; (b) at least one of the following: positive neostigmine test, decrement on low-frequency repetitive nerve stimulation, or positive serum antibodies; and (c) exclusion of other mimics (10). Immunotherapy-naive OMG patients treated initially with TAC or PRED monotherapy at our institution between January 2021 and September 2024 were included in this study. We excluded patients who were younger than 18 years, scored 0 or 1 on the Myasthenia Gravis Activities of Daily Living (MG-ADL) scale (11), had a follow-up duration of less than 12 months, used immunosuppressive agents other than TAC and PRED during the 12-month follow-up period, or had incomplete follow-up data. Finally, we identified 27 patients with TAC monotherapy and 15 patients with PRED monotherapy.

### Therapeutic regimens

Patients (n = 27) in the TAC group were treated with an initial dose of 1 mg twice a day and then adjusted the dose or added Wuzhi capsule (12) to reach a satisfactory serum concentration (4.8–9.0 ng/ml) (9, 13). Among the 27 patients receiving TAC monotherapy, the mean maximum daily dose was 2.11 ± 0.42 mg/day, and the mean maintenance daily dose at 12 months was 1.56 ± 0.71 mg/day. TAC blood concentrations were monitored at 1, 6, and 12 months after baseline, with mean levels of 6.32, 5.79, and 6.91 ng/mL, respectively, all within the effective therapeutic range.

In the PRED group, fifteen patients received oral PRED initially at 25–50 mg daily (median 30 mg). The dose was tapered by 5 mg every 1–2 weeks after symptom control, then by 2.5 mg every 2–4 weeks once ≤ 20 mg was reached. At the 12-month follow-up, nine patients remained on a daily dose of 5–15 mg, while six received 17.5–20 mg per day.

Neither group received any other immunosuppressants; cholinesterase inhibitors were allowed. Pyridostigmine tablets (60 mg/tablet) were initiated at 60 mg three times daily (180 mg/day) and individually adjusted according to symptom control and tolerability. Moreover, O-QMG assessments were conducted 6–8 h after pyridostigmine administration to avoid the potential influence of anticholinesterases.

### Data collection and outcome measurement

Information including sex, age at onset, disease duration, presenting symptoms, past medical history, seropositivity for anti-AChR, scores for MG-ADL and Quantitative Myasthenia Gravis (QMG) (14), and thymic status was collected. Ocular symptom severity was assessed using the MG-ADL and ocular Quantitative Myasthenia Gravis (O-QMG) scores. The O-QMG score, which specifically evaluates ptosis, diplopia, and facial muscle strength (range 0–9), was used as a key outcome to provide a more objective and ocular-focused assessment of treatment response, complementing the patient-reported MG-ADL (15). For the MG-ADL, we focused on the ocular-related items (ptosis and diplopia) in our OMG cohort.

The primary outcome was the time to reach minimum symptom expression (MSE), defined as MG-ADL ≤ 1 (16). As an exploratory endpoint, we also assessed the time to achievement of an MG-ADL score of 0. Secondary outcomes included: (1) the changes in MG-ADL and O-QMG scores from baseline (the initial dose of TAC or PRED) to month 1, 6, 12 after treatment, respectively; (2) the proportions of monotherapy retention patients (defined as consistent adherence to a single immunosuppressive agent) at month 12. The MSE definition was extrapolated from the generalized MG (GMG) literature, as no OMG-specific MSE definition is currently available. Safety was assessed based on medical records during hospitalization to monitor adverse events (AEs) and serious AEs (SAEs). Severity of AEs was graded according to the Common Terminology Criteria for Adverse Events (CTCAE) Version 5.0 (17).

### Statistical analyses

Statistical analyses were performed using SPSS version 27.0 (IBM Corp., USA), and data visualization was conducted with GraphPad Prism version 9.0 (GraphPad Software, USA). Normally distributed continuous variables were expressed as the mean ± standard deviation (SD), whereas nonnormally distributed continuous variables were presented as the median with interquartile range (IQR). The Shapiro–Wilk test was used to assess normality, and Levene’s test was applied to evaluate homogeneity of variance. Baseline intergroup comparisons were performed via independent-samples t tests for parametric data or Mann–Whitney U tests for nonparametric data. Categorical variables were presented as frequencies and percentages, with group differences evaluated using Fisher’s exact test. Time-to-event outcomes were compared using Kaplan-Meier curves with log-rank tests, and hazard ratios (HRs) with 95% confidence intervals (CIs) were derived from univariable Cox regression. Patients without the event were right-censored at last follow-up. A multivariable Cox model adjusted for baseline MG-ADL score and antibody status was used to assess the association with event attainment.

Changes in O-QMG and MG-ADL score from baseline to each post-baseline visit were analyzed using a restricted maximum likelihood (REML)-based mixed-effects model with repeated measures (MMRM). The model included treatment group, time point, and their interaction as fixed effects, with adjustment for the corresponding baseline score and antibody status. A missing-at-random (MAR) mechanism was assumed. An appropriate covariance structure was selected based on the lowest AIC (Akaike Information Criterion) or BIC (Bayesian Information Criterion) value (**Supplementary Table 5**). Bonferroni correction was applied to *post-hoc* pairwise comparisons across post-baseline time points in the MMRM analyses. To address the potential bias arising from the non-randomized design, we adjusted for covariates and performed a sensitivity analysis using the complete dataset while retaining patients in their initial treatment group. A two-tailed *P* value < 0.05 was considered statistically significant.

## Results

### Demographic and clinical features

We identified 27 and 15 patients in the TAC group and PRED group, respectively (**Figure 1**). The mean age at onset for all participants was 55.21 ± 11.96 years and 26.19% (11/42) were female. The median disease duration of all participants was 2 (1, 4) months. The demographic and clinical characteristics at baseline of 42 OMG patients are summarized in **Table 1**. Baseline characteristics, including sex, age at onset, disease duration, presenting symptoms, past medical history, anti-AChR seropositivity, MG-ADL and QMG scores, and thymic status, were similar between the mono-TAC and mono-PRED groups. No statistically significant difference in pyridostigmine use was observed between the PRED and TAC groups. The usage rate was 14/15 (93.3%) vs. 23/27 (85.2%), *P* = 0.539; the median starting dose was 180 mg/d (range 90–180) in both groups, and the median final dose was 180 mg/d (range 90–180) vs. 90 mg/d (range: 90–180), *P* = 0.208.

**Figure 1:**
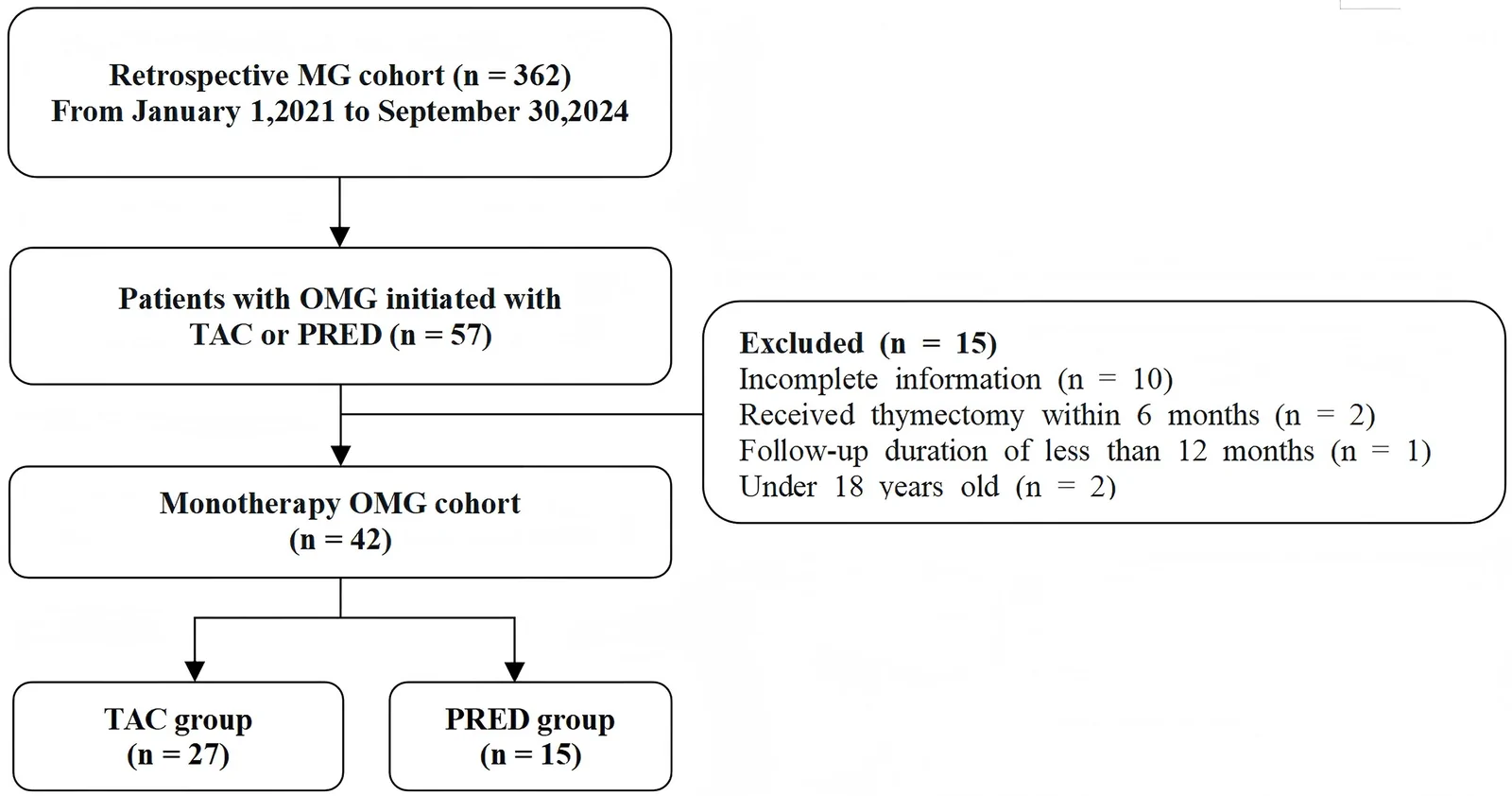
The workflow for enrollment in both treatment groups. OMG, ocular myasthenia gravis; TAC, tacrolimus; PRED, prednisone.

**Table 1:** Baseline characteristics of TAC group and the PRED group.

Characteristics	Initial monotherapy group			Full-course monotherapy group		
	TAC group (n = 27)	PRED group (n = 15)	*P* value	TAC group (n = 22)	PRED group (n = 11)	*P* value
Age at onset, years, mean ± SD	57.78 ± 10.35	50.60 ± 13.57	0.061	58.00 ± 10.25	52.00 ± 13.84	0.169
Disease course, month, median (IQR)	2 (0.5, 4.5)	2 (1.0,19.5)	0.192	2 (1.0,3.0)	2 (1.5,19.5)	0.233
Gender (male), n (%)	20 (74.1)	11 (73.3)	1.000	16 (72.7)	9 (81.8)	0.687
Early-onset MG^a^, n (%)	6 (22.2)	5 (33.3)	0.481	5 (22.7)	4 (36.4)	0.438
AChR-Ab positive, n (%)	17 (63.0)	11 (73.3)	0.734	15 (68.2)	8 (72.7)	1.000
Presenting symptom, n (%)						
Ptosis	21 (77.8)	14 (93.3)	0.390	16 (72.7)	10 (90.9)	0.378
Diplopia	9 (33.3)	5 (33.3)	1.000	8 (36.4)	3 (27.3)	0.709
Ptosis combined with diplopia	4 (14.8)	4 (26.7)	0.425	3 (13.6)	2 (18.2)	1.000
Past medical history, n (%)						
Hypertension	10 (37.0)	3 (20.0)	0.314	9 (40.9)	3 (27.3)	0.703
Diabetes	3 (11.1)	3 (20.0)	0.649	2 (9.1)	1 (9.1)	1.000
Coronary artery disease	3 (11.1)	0 (0.0)	0.541	2 (9.1)	0 (0.0)	0.542
Baseline MG-ADL score, mean ± SD	3.67 ± 1.14	4.13 ± 1.41	0.250	3.45 ± 0.91	4.09 ± 1.51	0.141
Baseline O-QMG score, mean ± SD	3.67 ± 1.33	4.20 ± 1.57	0.250	3.45 ± 1.22	4.27 ± 1.62	0.114
Thymoma, n (%)	2 (7.4)	2 (13.3)	0.608	2 (9.1)	1 (9.1)	1.000
Max daily dose, mean ± SD (mg/d)	2.11 ± 0.42	33.67 ± 9.57	–	2.01 ± 0.56	33.31± 9.01	–
Maint daily dose, mean ± SD (mg/d)	1.56 ± 0.71	18.67 ± 7.34	–	1.61 ± 0.44	18.97 ± 7.00	–

TAC, tacrolimus; PRED, prednisone; AChR-Ab, acetylcholine receptor antibodies; MG-ADL, Myasthenia Gravis Activities of Daily Living; O-QMG, ocular Quantitative Myasthenia Gravis; SD, standard deviation; IQR, interquartile range.

^a^ Onset age was younger than 50 years old.

### Immunotherapy treatment response

During the 12-month follow-up, one patient (1/27, 3.7%) in the TAC group developed GMG after voluntarily discontinuing TAC; no patient in the PRED group developed GMG (0/15).

Kaplan-Meier analysis with the primary endpoint of MSE (MG-ADL ≤ 1) showed that the median time to MSE attainment was 6.0 months (95% CI: 2.523, 9.477) for the PRED group and 12.0 months (95% CI: 8.342, 15.658) for the TAC group. The Log-rank test revealed no statistically significant difference between the two groups (χ² = 1.306, *P* = 0.253). In a Cox regression model adjusted for baseline MG-ADL score and antibody status to control for potential confounding, the HR for MSE attainment in the TAC group compared with the PRED group was 1.757 (95% CI: 0.740, 4.172, *P* = 0.202). Similarly, no statistically significant difference was observed in the median time to achieve an MG-ADL score of 0 between the two groups. (log-rank *P* = 0.928; adjusted HR = 0.815, *P* = 0.735) (**Figure 2**).

**Figure 2:**
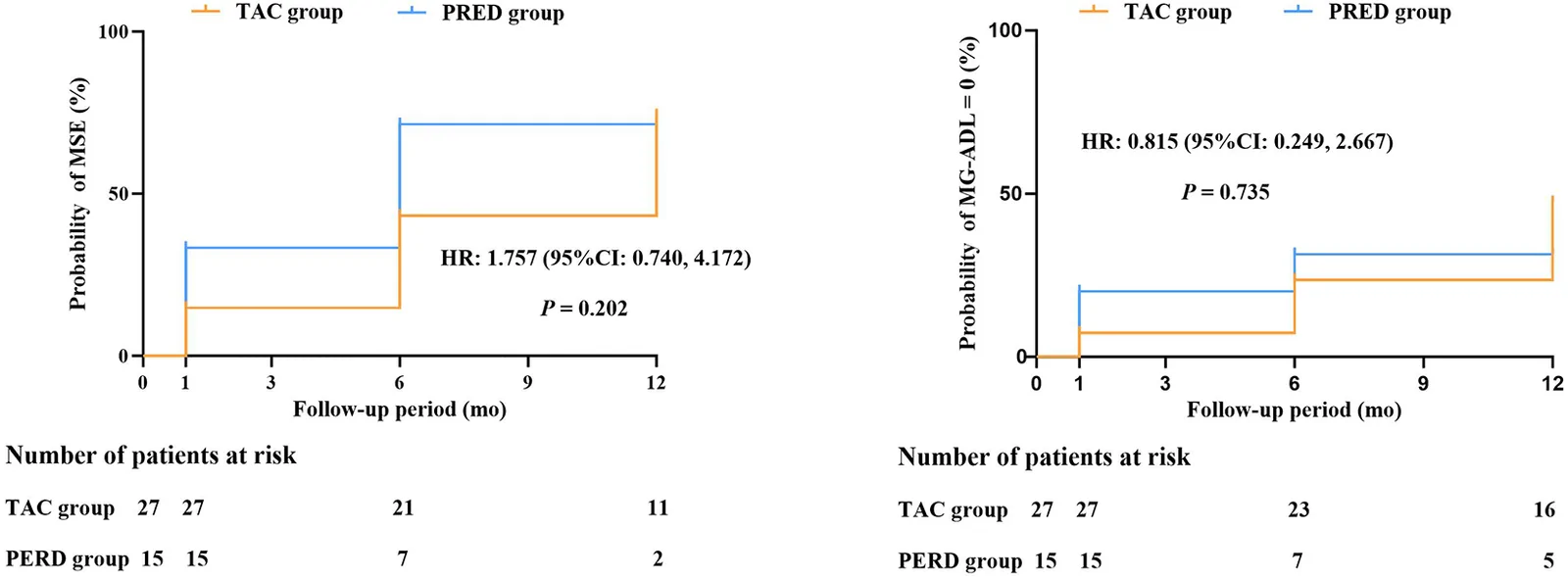
Kaplan-Meier curves for time to MSE attainment, MG-ADL = 0, and number at risk. MSE was defined as MG-ADL ≤ 1. The number at risk at each time point, shown below the x-axis, reflects patients still at risk before that time point. Hazard ratios (HRs) with 95% confidence intervals (CIs) and *P* values were derived from adjusted Cox proportional hazards regression, with the PRED group as the reference. TAC, tacrolimus; PRED, prednisone; MSE, minimum symptom expression.

For secondary outcomes, MMRM analyses showed no significant group-by-time interaction for MG-ADL (*F* = 0.630, *P* = 0.600) or O-QMG scores (*F* = 1.656, *P* = 0.181), nor any significant overall group effect for either measure (MG-ADL: *F* = 0.182, *P* = 0.672; O-QMG: *F* = 0.768, *P* = 0.385). However, significant main effects of time were observed for both measures (MG-ADL: *F* = 14.686, *P* < 0.001; O-QMG: *F* = 11.519, *P* < 0.001), indicating that both groups improved significantly over the 12-month follow-up, with comparable trajectories between the TAC and PRED groups.

The least squares means (LS means) changes in MG-ADL and O-QMG scores during follow-up in both treatment groups are shown in **Table 2**. For the MG-ADL score, *post-hoc* contrasts with Bonferroni correction showed no significant between-group differences in changes from baseline at any time point (all *P* > 0.05; **Figure 3A**). For the O-QMG score, a significant between-group difference was observed only at month 6 (Δ = 1.573, 95% CI: 0.080, 3.065, *P* = 0.039), favoring the PRED group (mean change: −2.737 vs. −1.164 for PRED and TAC, respectively), suggesting a more rapid improvement in the PRED group. No significant differences were found at month 1 or month 12 (both *P* > 0.05; **Figure 3B**). Temporal changes in the ADL and O-QMG scores for all individual cases are shown in the **Figure 4**. Sensitivity analyses using the complete dataset, which retained patients in their initial treatment group, yielded results that were not substantially different from the primary analysis. (**Supplementary Tables 1**, **2**; **Supplementary Figure 1**). In addition, the sensitivity analyses included comparisons of ocular MG-ADL score changes and time to ocular MG-ADL = 0 (**Supplementary Table 3**; **Supplementary Figure 2**).

**Table 2:** The least squares means (95% CI) changes from baseline in MG-ADL and O-QMG scores during follow-up in both treatment groups.

Clinical scores		TAC (n = 27)	PRED (n = 15)	Between-group difference	*P* value
Baseline	MG-ADL LS mean (95% CI)	-	-	-	-
	O-QMG LS mean (95% CI)	-	-	-	-
1m	MG-ADL LS mean (95% CI)	−0.704 (−1.721, 0.313)	−1.333 (−2.698, 0.031)	0.629 (−0.609, 1.869)	0.311
	O-QMG LS mean (95% CI)	−0.630 (−1.658, 0.399)	−1.267 (−2.647, 0.113)	0.637 (−0.631, 1.905)	0.381
6m	MG-ADL LS mean (95% CI)	−1.836 (−2.871, −0.802)	−2.379 (−3.817, −0.941)	0.543 (−0.748, 1.833)	0.401
	O-QMG LS mean (95% CI)	−1.164 (−2.340, 0.012)	−2.737 (−4.378, −1.096)	1.573 (0.080, 3.065)	0.039^*^
12m	MG-ADL LS mean (95% CI)	−2.066 (−3.602, −0.529)	−1.940 (−4.113, 0.232)	−0.126 (−2.054, 1.803)	0.895
	O-QMG LS mean (95% CI)	−1.871 (−3.123, −0.620)	−2.307 (−4.037, −0.577)	0.436 (−1.143, 2.015)	0.586

Data are presented as LS mean change (95% CI). The columns “TAC” and “PRED” show the within-group change from baseline at each time point. The column “Between-group difference” represents the difference between these two within-group changes (TAC-PRED). P values were derived from the MMRM model, with adjustment for baseline score and antibody status, and Bonferroni correction for multiple comparisons. At 6 months, the between-group difference in O-QMG change from baseline was statistically significant; TAC, tacrolimus; PRED, prednisone; CI, confidence interval; *P < 0.05.

**Figure 3:**
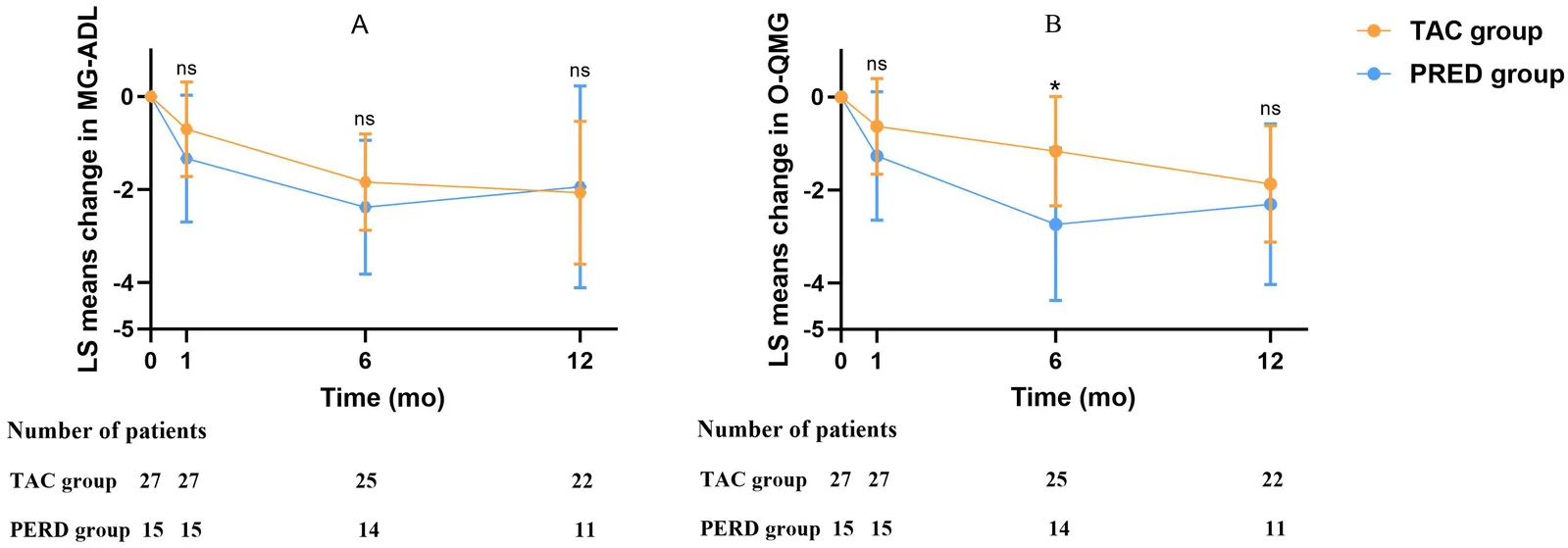
The least squares means (95% CI) changes from baseline in MG-ADL **(A)** and O-QMG **(B)** scores during follow-up in both treatment groups. The number of patients at each time point is indicated below the x-axis. Error bars represent 95% confidence intervals. **(A)** *Post-hoc* contrasts with Bonferroni correction showed no significant between-group differences in MG-ADL changes from baseline at any time point. **(B)** For O-QMG, a significant difference in the change from baseline was observed at month 6, and no significant differences were found at month 1 or month 12. MG-ADL, Myasthenia Gravis Activities of Daily Living; O-QMG, ocular Quantitative Myasthenia Gravis; TAC, tacrolimus; PRED, prednisone; *P < 0.05; ns, not significant between groups.

**Figure 4:**
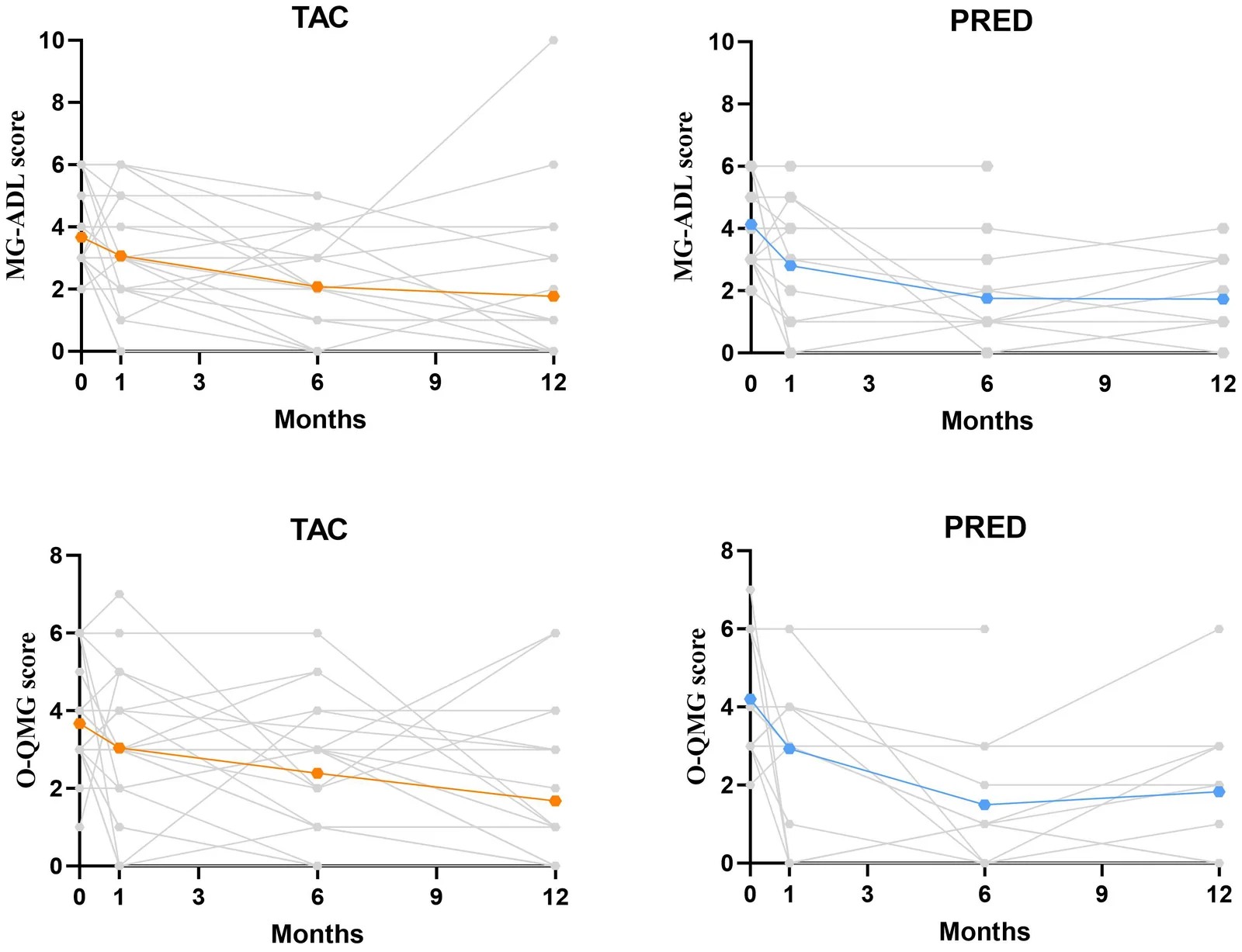
Temporal changes in the MG-ADL and O-QMG scores for individual cases are shown in the graphs. Gray lines represent individual patient trajectories of actual observed scores. Colored lines indicate the group-specific least squares (LS) mean scores (yellow for TAC and blue for PRED), estimated from the mixed-effects model with repeated measures (MMRM), with adjustment for baseline score and antibody status. MG-ADL, Myasthenia Gravis Activities of Daily Living; O-QMG, ocular Quantitative Myasthenia Gravis; TAC, tacrolimus; PRED, prednisone.

The proportions of monotherapy retention patients were comparable between the TAC and the PRED groups at month 12 (81.5% vs. 73.3%, *P* = 0.698).

### Safety

Six patients (22.2%) in the TAC group and 4 patients (26.7%) in the PRED group had adverse drug reactions during the study. In the TAC group, the observed adverse reactions included diarrhea in 2 cases (7.4%), upper respiratory tract inflammation in 1 case (3.7%), muscle spasm in 1 case (3.7%), and blood glucose increased in 2 cases (7.4%), all of which were relieved after symptomatic treatment. In the PRED group, the adverse reactions included blood glucose increased in 3 cases (20.0%) and hypertension in 1 case (6.7%), which also resolved following symptomatic management. There was no statistically significant difference in the overall incidence of adverse reactions between the two groups (*P* = 1.000).

## Discussion

In the present study, mono-TAC provided improvement with an acceptable safety profile in patients with newly diagnosed OMG, suggesting that it may serve as a viable treatment option for this population. The median time to MSE attainment showed only a modest difference between the TAC and PRED groups, which did not reach statistical significance during follow-up.

OMG is not life-threatening and is generally considered a mild disease; however, diplopia and ptosis often cause troublesome limitations in activities of daily living and impair quality of life (18). The natural history of OMG is highly variable; although some patients remain purely ocular, 50–80% progress to GMG, with the peak risk occurring during the first 1–2 years after onset—a finding that underscores the critical window for early intervention (19).

Early immunotherapeutic intervention at the ocular stage may suppress disease progression to GMG (20). Oral PRED is the most commonly used and effective treatment for OMG (20). TAC, as a nonsteroidal immunosuppressant, has a beneficial effect in the treatment of MG (21–23). TAC is usually used in combination with corticosteroids and acts as a steroid-sparing agent to reduce the corticosteroid burden in patients. However, patients still require long-term corticosteroid therapy, which carries the risk of steroid-related adverse effects (24). TAC as monotherapy has been investigated in other immune-mediated diseases, including adult-onset minimal change nephrotic syndrome (25) and refractory ulcerative colitis (26). In China, TAC monotherapy is commonly used in clinical practice for OMG. However, evidence-based medical data regarding the efficacy of mono-TAC in adult OMG remains scarce, with most relevant studies being case reports (27).

The largest retrospective study of mono-tacrolimus in MG included 34 patients with Myasthenia Gravis Foundation of America (MGFA) class I disease but did not report OMG-specific outcomes (28). Another study also reported that mono-TAC is effective in mild-to-moderate MG, with better tolerability and similar efficacy compared with PRED (23). Interestingly, it appears to achieve faster remission in OMG (41 patients) than in GMG (57 patients). Similarly, in the present study, the 12-month KM-estimated cumulative incidence of MSE attainment was 74.2% in the TAC group and 71.4% in the PRED group. A summary of the immunotherapy treatment response in both groups is provided in **Supplementary Table 4**. Collectively, these findings indicate that mono-TAC could be considered as a treatment option for OMG, warranting further investigation in larger prospective studies.

Our study revealed a discernible temporal pattern in treatment response between the two groups. In the early phase (1 to 6 months), patients in the PRED group achieved a numerically higher rate of MSE attainment, and also demonstrated a greater change from baseline in O-QMG scores at month 6 than the TAC group, which likely reflects the rapid anti-inflammatory action characteristic of PRED. In contrast, the therapeutic onset of TAC appeared slower, with a cumulative MSE attainment rate of only 14.8% at 1 month. However, a potential ‘catch-up’ effect was observed at the 12-month mark; this aligns with prior studies that have reported long-term efficacy of TAC (29). Accordingly, the cumulative MSE attainment rate in the TAC group (74.2%) was numerically higher than that of the PRED group (71.4%). Although this numerical difference did not reach statistical significance, possibly due to the limited sample size, no conclusion regarding equivalence can be drawn. These preliminary findings suggest that although the onset of TAC is slower, it may offer a viable option for the sustained management of OMG patients.

In the PRED group, four patients added TAC due to persistent symptoms, initiated at month 1 (n = 2), month 4 (n = 1), and month 7 (n = 1). At 12 months, their median MG-ADL score had decreased by 2 points from the time of TAC initiation (*P* = 0.066). Although this change did not reach statistical significance, it may suggest a potential clinical benefit of add-on TAC in patients with suboptimal response to mono-PRED. Furthermore, although the present study did not directly assess steroid dose reduction, previous studies have shown that TAC acts as a steroid-sparing agent, suggesting its potential role as a rescue or adjunctive therapy (21, 29–32). At the end of the 12-month follow-up period, the monotherapy retention rate in the TAC group was numerically higher than that in the PRED group, though the difference was not statistically significant. Nevertheless, the numerical difference in retention rate should be interpreted with caution given the small sample size.

While PRED is frequently prescribed, it is not without drawbacks. Long-term PRED use can result in various side effects, including osteoporosis, weight gain, acne, elevated blood pressure, mood fluctuations, and a cushingoid appearance (33). It has also been linked to rare complications such as gastric and esophageal irritation, compressive fractures, and aseptic femoral head necrosis (34). Furthermore, the adverse effects of PRED are dose-dependent, with higher dosages being significantly associated with an increased number of comorbidities and AEs, such as osteoporosis and fractures, among others. (35, 36). A critical finding in our study is the residual steroid burden at the end of the follow-up period. Despite achieving clinical stability, 6 patients (40%) in the PRED group remained on a daily dose exceeding 15 mg per day. This finding highlights the substantial burden of clinical remission in the PRED group. Prolonged exposure to moderate doses of PRED significantly elevates the risk of cumulative systemic toxicities. The clinical relevance of persistent high-dose steroid use cannot be overlooked, as it translates directly into a higher cumulative risk for complications. In contrast, the favorable safety profile of TAC in the treatment of MG has been demonstrated in multiple studies (22, 29, 37). A subset of patients may develop adverse effects from TAC, including hypertension, nephrotoxicity, and various neuropsychiatric problems (8). Consistently, only a few adverse events were observed in the present study, all of which were mild and reversible. Moreover, case reports and retrospective analyses had also shown favorable safety of TAC in pediatric MG populations (38–40). Taken together, these findings suggest the role of TAC as a reasonable therapeutic alternative in the management of OMG.

Mechanistically, although TAC and PRED act via distinct pathways, they ultimately converge on the same core pathogenic process of MG–the T-cell-dependent humoral immune response. PRED reduces lymphocyte counts and promotes regulatory T-cell differentiation, whereas TAC inhibits calcineurin to block T-cell activation and subsequent auto-antibody production (41). Both agents effectively interfere with T–B cell cooperation, thereby attenuating the autoimmune cascade.

In our monotherapy cohort, a subset of patients still failed to achieve MSE, highlighting the inherent limitations of single-agent therapy. For those with suboptimal responses, combined therapy with PRED and TAC may be attempted. Bi et al. reported that in pediatric-onset OMG patients who failed to respond to 3 months of steroid monotherapy, TAC add-on therapy could alleviate symptoms (42).

This study has several limitations. First, the sample size was relatively small, which may have contributed to the lack of statistically significant differences in MG-ADL and O-QMG score changes between the two groups, and may also have increased sampling error. Second, this study was retrospective and non-randomized. Residual confounding cannot be fully excluded despite adjustment for baseline MG-ADL score and antibody status, and other factors such as time-varying pyridostigmine use may also have influenced symptom scores; in addition, the MAR assumption underlying the MMRM may not hold, potentially overestimating treatment efficacy. Third, there is currently no validated outcome measure specifically designed for OMG. Although we used a combination of MG-ADL and O-QMG to capture both patient-reported and objective ocular symptoms, these instruments were originally developed for GMG and may not fully capture the spectrum of ocular involvement. This may have led to incomplete assessment of treatment response. In addition, the study was not powered to assess whether TAC or PRED prevents generalization from ocular to generalized MG, and no causal inference regarding the single generalization event can be drawn. Future studies with larger sample sizes, longer follow-up, and OMG-specific outcome measures are warranted to confirm our findings.

## Conclusion

In conclusion, no statistically significant difference was observed in treatment response between TAC and PRED monotherapy in OMG. However, the study was not designed as an equivalence or non-inferiority trial, and no conclusion regarding equivalence can be drawn. TAC may serve as a reasonable alternative for patients who cannot tolerate or refuse steroids. Larger studies are needed to confirm these findings.

## Data Availability

The raw data supporting the conclusions of this article will be made available by the authors, without undue reservation.

## Supplementary material

Supplementary Figure 1 Sensitivity analysis using the complete dataset: Kaplan-Meier curves for time to MSE attainment and MG-ADL = 0, and number at risk. MSE was defined as MG-ADL ≤ 1. The number at risk at each time point, shown below the x-axis, reflects patients still at risk before that time point. Hazard ratios (HRs) with 95% confidence intervals (CIs) and *P* values were derived from adjusted Cox proportional hazards regression, with the PRED group as the reference. TAC, tacrolimus; PRED, prednisone; MSE, minimum symptom expression.

Supplementary Figure 2 Kaplan-Meier curves for time to ocular MG-ADL = 0 and number at risk. The ocular MG-ADL subscore includes ptosis and diplopia, with a total score of 6. The number at risk at each time point, shown below the x-axis, reflects patients still at risk before that time point. Hazard ratios (HRs) with 95% confidence intervals (CIs) and *P* value was derived from adjusted Cox proportional hazards regression, with the PRED group as the reference. TAC, tacrolimus; PRED, prednisone.
