# Supplementary material for: Comparison between mono-tacrolimus and mono-prednisone in the treatment of ocular myasthenia gravis: a retrospective, observational cohort study
Source: Front Immunol. 2026 Oct 13;17:1917837. doi: 10.3389/fimmu.2026.1917837 (PMC13710400; doi:10.3389/fimmu.2026.1917837)
Supplement: Supplementary file 1 [file SupplementaryFile1.docx]

Supplement Table 1. Sensitivity analysis using the complete dataset (including excluded cases): fixed effects tests from the MMRM model for MG-ADL and O-QMG scores.

| Dependent Variable | Tests of Type III Fixed Effects | | | | | |
| --- | --- | --- | --- | --- | --- | --- |
|  | Source | Df1 | | Df2 | *F* | *P* value |
| MG-ADL | Group | 1 | 37.128 | | 0.141 | 0.710 |
|  | Time | 3 | 40.000 | | 13.857 | 0.000 |
|  | Group × Time | 3 | 40.000 | | 0.402 | 0.752 |
|  | Baseline_ADL | 1 | 38.000 | | 239.953 | 0.000 |
|  | Antibody | 1 | 38.000 | | 1.366 | 0.250 |
| O-QMG | Group | 1 | 51.185 | | 0.031 | 0.861 |
|  | Time | 3 | 112.894 | | 10.869 | 0.000 |
|  | Group × Time | 3 | 112.894 | | 0.965 | 0.412 |
|  | Baseline_ADL | 1 | 53.992 | | 7.464 | 0.008 |
|  | Antibody | 1 | 53.992 | | 0.137 | 0.713 |

Abbreviations: Df1, numerator degrees of freedom; Df2, denominator degrees of freedom; MG-ADL, Myasthenia Gravis Activities of Daily Living; O-QMG, ocular Quantitative Myasthenia Gravis.

Supplement Table 2. Sensitivity analysis using the complete dataset (including excluded cases): the least squares means (95% CI) changes from baseline in MG-ADL and O-QMG scores during follow-up in both treatment groups.

| Clinical scores | | TAC (n = 27) | PRED (n = 15) | Between-group difference | *P* value |
| --- | --- | --- | --- | --- | --- |
| Baseline | MG-ADL LS mean (95% CI) | - | - | - | - |
|  | O-QMG LS mean (95% CI) | - | - | - | - |
| 1m | MG-ADL LS mean (95% CI) | −0.704 (−1.721, 0.313) | −1.333 (−2.698, 0.031) | 0.629 (−0.609, 1.869) | 0.311 |
|  | O-QMG LS mean (95% CI) | −0.630 (−1.658, 0.399) | −1.267 (−2.647, 0.114) | 0.637 (−0.633, 1.907) | 0.322 |
| 6m | MG-ADL LS mean (95% CI) | −1.778 (−2.811, −0.745) | −2.067 (−3.453, −0.681) | 0.289 (−0.970, 1.548) | 0.645 |
|  | O-QMG LS mean (95% CI) | −1.185 (−2.349, −0.022) | −2.200 (−3.761, −0.639) | 1.015 (−0.424, 2.454) | 0.166 |
| 12m | MG-ADL LS mean (95% CI) | −2.222 (−3.638, −0.806) | −2.200 (−4.100, −0.300) | −0.022 (−1.747, 1.703) | 0.979 |
|  | O-QMG LS mean (95% CI) | −2.000 (−3.202, −0.798) | −2.133 (−3.746, −0.521) | 0.133 ( -1.353, 1.620) | 0.860 |

Legend: Data are presented as LS mean change (95% CI). The columns "TAC" and "PRED" show the within-group change from baseline at each time point. The column "Between-group difference" represents the difference between these two within-group changes (TAC-PRED). *P* values were derived from the MMRM model, with adjustment for baseline score and antibody status, and Bonferroni correction for multiple comparisons. TAC, tacrolimus; PRED, prednisone; CI, confidence interval; *: *P* < 0.05.

Supplement Table 3. The least squares means (95% CI) changes from baseline in Ocular MG-ADL scores during follow-up in both treatment groups.

| Clinical scores | TAC (n = 27) | PRED (n = 15) | Between-group difference | P value |
| --- | --- | --- | --- | --- |
| Baseline | - | - | - | - |
| 1m | −0.778 (−1.616, 0.060) | −1.667 (−2.791, -0.542) | 0.889 (−0.145, 1.923) | 0.091 |
| 6m | −2.035 (−3.034, −1.036) | −2.456 (−3.850, −1.061) | 0.421 (−0.847, 1.689) | 0.513 |
| 12m | −2.499 (−3.584, −1.414) | −2.337 (−3.839, −0.834) | −0.162 (−1.532, 1.207) | 0.814 |

Legend: Data are presented as LS mean change (95% CI). The columns "TAC" and "PRED" show the within-group change from baseline at each time point. The column "Between-group difference" represents the difference between these two within-group changes (TAC-PRED). P values were derived from the MMRM model, with adjustment for baseline score and antibody status, and Bonferroni correction for multiple comparisons. TAC, tacrolimus; PRED, prednisone; CI, confidence interval; *: *P* < 0.05.

Supplement Table 4. Summary of immunotherapy treatment response in both groups.

| Outcome | TAC group (n = 27) | PRED group (n = 15) | *P* value |
| --- | --- | --- | --- |
| Primary outcome |  |  |  |
| Median time to MSE, months (95% CI) | 12.0 (8.342, 15.658) | 6.0 (2.523, 9.477) | 0.253 |
| HR (95% CI) | 1.757 (0.740, 4.172) | Reference | 0.202 |
| Exploratory endpoint |  |  |  |
| Median time to MG-ADL = 0, months (95% CI) | - | - | 0.928 |
| HR (95% CI) | 0.815 (0.249, 2.667) | Reference | 0.735 |
| Disease progression |  |  |  |
| Progression to GMG, n (%) | 1 (3.7%) | 0 (0%) | 1.000 |

Legend: Data are presented as median (95% CI), n (%), or HR (95% CI). MSE was defined as MG-ADL ≤ 1. Time-to-event outcomes were estimated using the Kaplan–Meier method. Hazard ratios (HRs) were derived from univariable Cox regression, with the PRED group as the reference. Median survival time could not be estimated for either group in O-QMG as the cumulative incidence did not exceed 50% during follow-up. *P* value was derived from the log-rank test. TAC, tacrolimus; PRED, prednisone; MSE, minimum symptom expression; GMG, generalized myasthenia gravis; CI, confidence interval; HR, hazard ratio.

Supplement Table 5. Model fit comparisons for different covariance structures

| Outcome | Covariance structure | No. of parameters | –2 Restricted log-likelihood | AIC | BIC |
| --- | --- | --- | --- | --- | --- |
| ADL | AR(1) | 2 | 568.55 | 572.55 | 578.489 |
|  | CS | 2 | 578.547 | 582.547 | 588.486 |
|  | **UN** | **10** | **498.495** | **518.495** | **548.194** |
| O-QMG | **AR(1)** | **2** | **575.581** | **579.581** | **585.521** |
|  | CS | 2 | 579.37 | 583.37 | 589.309 |
|  | UN | 10 | -11.658 | 8.342 | 38.04 |

AIC, Akaike information criterion; BIC, Bayesian information criterion; AR(1), first-order autoregressive; CS, compound symmetry; UN, unstructured；O-QMG, ocular myasthenia gravis quality of life

Note: The model with the lowest AIC and BIC values was selected as the final model for each outcome. For ADL, UN was selected; for O-QMG, AR (1) were selected (UN did not converge). The smallest values for each outcome are indicated in bold.

Supplement Figure 1. Sensitivity analysis using the complete dataset: Kaplan-Meier curves for time

to MSE attainment, MG-ADL = 0, and number at risk.

Legend: Sensitivity analysis using the complete dataset: Kaplan-Meier curves for time to MSE attainment, MG-ADL = 0, and number at risk. MSE was defined as MG-ADL ≤ 1. The number at risk at each time point, shown below the x-axis, reflects patients still at risk before that time point. Hazard ratios (HRs) with 95% confidence intervals (CIs) and *P* values were derived from adjusted Cox proportional hazards regression, with the PRED group as the reference. TAC, tacrolimus; PRED, prednisone; MSE, minimum symptom expression.

Supplement Figure 2. Kaplan-Meier curves for time to Ocular MG-ADL = 0 and number at risk.

Legend: Kaplan-Meier curves for time to Ocular MG-ADL = 0 and number at risk. The Ocular MG-ADL subscore includes ptosis and diplopia, with a total score of 6. The number at risk at each time point, shown below the x-axis, reflects patients still at risk before that time point. Hazard ratios (HRs) with 95% confidence intervals (CIs) and *P* value was derived from adjusted Cox proportional hazards regression, with the PRED group as the reference. TAC, tacrolimus; PRED, prednisone.
